# C-phycocyanin improves the developmental potential of cryopreserved human oocytes by minimizing ROS production and cell apoptosis

**DOI:** 10.1371/journal.pone.0300538

**Published:** 2024-04-01

**Authors:** Lu Wang, Hao-Ran Liu, Teng Wang, Meng-Lei Feng, Zhao-Yu Jiang, Qi Yang, Dui Sun, Chun-Ru Song, Xiu-Juan Zhang, Cheng-Guang Liang

**Affiliations:** 1 State Key Laboratory of Reproductive Regulation & Breeding of Grassland Livestock, Institutes of Biomedical Sciences, School of Life Sciences, Inner Mongolia University, Hohhot, Inner Mongolia, People’s Republic of China; 2 Department of Obstetrics and Gynecology, Reproductive Medicine Center, Inner Mongolia Baogang Hospital, Baotou, Inner Mongolia, People’s Republic of China; 3 Inner Mongolia Academy of Science and Technology, Hohhot, Inner Mongolia, People’s Republic of China; University of Massachusetts Amherst, UNITED STATES

## Abstract

**Purpose:**

The cryopreservation process damages oocytes and impairs development potential. As a potent antioxidant, C-phycocyanin (PC) regulates reproductive performance. However, its beneficial effects on vitrified human oocytes remain unknown.

**Methods:**

In this study, human GV-stage oocytes obtained from controlled ovarian hyperstimulation (COH) cycles were randomly allocated to three groups: fresh oocyte without freezing (F group), vitrification in medium supplemented with PC (P group), and vitrification in medium without PC as control group (C group). After warming, viable oocytes underwent *in vitro* maturation.

**Results:**

Our results showed that 3 μg/mL PC treatment increased the oocyte maturation rate after cryopreservation. We also found that PC treatment maintains the regular morphological features of oocytes. After PC treatment, confocal fluorescence staining showed a significant increase in the mitochondrial membrane potential of the vitrified oocytes, along with a notable decrease in intracellular reactive oxygen species and the early apoptosis rate. Finally, after *in vitro* maturation and parthenogenetic activation, vitrified oocytes had a higher potential for cleavage and blastocyst formation after PC treatment.

**Conclusion:**

Our results suggest that PC improves the developmental potential of cryopreserved human GV-stage oocytes by attenuating oxidative stress and early apoptosis and increasing the mitochondrial membrane potential.

## Introduction

In assisted reproductive technology for humans, oocyte cryopreservation is essential for fertility preservation, especially for patients indicated to undergo cancer chemoradiotherapy [1]. In recent years, there have been significant improvements in the protocols used for mature oocyte cryopreservation, leading to better survival outcomes, fertilization, and embryo implantation [2–4]. However, freezing immature oocytes is more practical for patients with contraindications to ovarian stimulation, such as ovarian hyperstimulation syndrome, or those who need urgent chemoradiotherapy [5]. In addition, germinal vesicle (GV)-stage oocytes lack temperature-sensitive and chemically sensitive meiotic spindles. Theoretically, cryopreservation of GV-stage oocytes avoids damage to the functional integrity of the meiotic spindle formed after germinal vesicle breakdown (GVBD), thereby preventing the occurrence of aneuploidy [6–8]. After thawing, the recovered GV-stage oocytes undergo *in vitro* maturation (IVM) and then *in vitro* fertilization and embryo transfer (IVF-ET).

Clinical studies have shown that GV-stage oocytes from different sources have different survival potentials, maturation levels, fertilization rates, and preimplantation developmental characteristics after thawing [9–17]. When the vitrification method is used, the survival rates of oocytes vitrified at the GV-stage versus MII-stage are not significantly different. Following insemination by intracytoplasmic sperm injection (ICSI), developmental rates are comparable, but high-quality embryo rates are lower [16]. Compared with oocytes subjected to vitrified freezing or obtained as fresh controls, oocytes treated with the slow freezing protocol are more likely to activate spontaneously after IVM, accompanied by higher spindle abnormality [18]. After IVM and fertilization of the resuscitated GV-stage oocytes, the maturation rate and developmental capacity become significantly lower than those obtained by vitrified MII oocytes [19]. Abnormal cytoplasm is the main factor influencing the effect of vitrification on GV-stage oocytes. One study on the ultrastructure of post-vitrification warmed IVM oocytes showed that the oocytes had generally rounded mitochondria with few peripheral or transverse cristae [20]. Since mitochondria provide ATP for oocytes at maturation and fertilization and their morphology and distribution are related to cell metabolism, proliferation, and differentiation levels, an insufficient number or abnormal morphology of mitochondria may not be able to satisfy the ATP requirements of the oocyte, resulting in a higher incidence of chromosomal aberrations [21,22]. Additionally, during the cryopreservation, reactive oxygen species (ROS) are produced, thereby increasing the risk of ROS induced impairment of cellular functions and survival. Antioxidants are a potential additive that have been reported to partially or completely reverse damage associated with freeze-thaw stress [23]. Numerous attempts have been made in the past decade to minimize cryoinjury. Antioxidants such as antifreeze proteins, selenium, superoxide dismutase (SOD), or melatonin have been added to the cryopreservation media to mitigate this effect, with varying degrees of success [24–27]. Recently, researchers have significantly improved the cryopreservation effect of human oocytes through adding melatonin, by inhibiting oxidative stress and maintaining membrane permeability [28]. While alternative strategies hold promise for improving oocyte survival and function after thawing, their application requires careful evaluation to avoid unintended consequences and to ensure the safety and efficacy of the cryopreservation process.

C-Phycocyanin (PC), a notable biliprotein, is obtained from Spirulina platensis and is predominantly found in red algae, cyanobacteria, and cryptophytes [29]. PC scavenges various types of free radicals, such as alkoxy, hydroxyl, and peroxyl radicals. It also reduces the production of nitrite, suppresses the expression of iNOS, and inhibits the peroxidation of microsomal lipids. PC, acting as an antioxidant, hinders the aging process and safeguards the functionality of mitochondria in various types of cells [30]. Our previous research demonstrated that the sustained intragastric administration of PC significantly reduced the accumulation of ROS in the oocytes of galactose-induced aging mice. Additionally, this treatment improved the quality of oocytes, leading to a boost in female reproductive capacity [31]. Recently, we reported that PC has the ability to improve the fertility of obese mice by reversing DNA damage, thereby enhancing the quality of ovaries and oocytes [32]. Furthermore, a growing body of evidence suggests that PC amplifies cellular functions, eliminates detrimental radicals, and improves organ performance. As a result, PC is widely used as a natural component with antioxidant, neuroprotective, anti-inflammatory, and oxygen free-radical scavenging properties [30,33].

A recent study proved that PCs were effective in promoting porcine embryonic development [34]. However, whether PC can improve the developmental competence of vitrified immature human oocytes is still unknown. Therefore, in this study, to compare the oocytes from the fresh control (F), vitrification control (C), and vitrification supplemented with PC in medium (P) groups, we evaluated cell survival, maturation, mitochondrial membrane potential (MMP), ROS, early apoptosis, and embryo development. We found that PC played a protective role against vitrification injury in human GV-stage oocytes by attenuating both oxidative stress and early apoptosis and increasing MMP. Our study provides a practical technical approach to improving the developmental rate of frozen oocytes in clinical practice.

## Materials and methods

### Ethics statement

The study was approved by the Committee of Medical Ethics of Inner Mongolia Baogang Hospital, China (Ethics approval number: 2020MER-003). Patients participating in the study received comprehensive information and signed on consent forms before the commencement of the oocyte retrieval procedure.

### Inclusion criteria

The study included patients under the age of 35 who underwent IVF-ICSI procedures from June 2020 to December 2022. The patient parameters for age, stimulation protocol, and numbers of mature, immature, and total oocytes retrieved were comparable among all three groups. Cycles involving endometriosis, hyporesponsiveness, and ovarian failure were excluded. Patients had at least two GV-stage oocytes, and the number of GV-stage oocytes did not exceed 50% of the cohort collected at retrieval with similar size and status of cumulus cells.

### Experimental design

The study was designed to evaluate the effects of PC on the cryopreservation of immature human oocytes. Obtaining GV-stage oocytes from unstimulated ovaries is difficult due to the scarcity of donated human ovariantissue for research. Comparable behavior is observed in immature oocytes from both stimulated and unstimulated cycles, leading to the formation of blastocysts with normal chromosomes [17]. Hence, this study utilized GV-stage oocytes unsuitable for ICSI cycles.

In experiment I, 367 GV-stage oocytes were randomly distributed into the following groups through a computer-generated list. Noncryopreserved 49 GV-stage oocytes were included in the fresh group (F group) of oocytes subjected to IVM to the MII stage. The other 318 GV-stage oocytes were included in the vitrification group and subjected to vitrification at the immature stage, thawing, and IVM to the MII stage.

To evaluate the effect of PC on cryopreservation, 64 GV-stage oocytes that did not receive any PC treatment were assigned to the control group (C group). The other 254 GV-stage oocytes were immersed in the equilibration solution (ES), vitrification solution (VS), thawing solution (TS), dilution solution (DS), warming solution (WS), IVM medium, and embryo culture medium. All the solutions or media were supplemented with different concentrations (1 μg/mL, 3 μg/mL, 5 μg/mL, and 7 μg/mL) of PC. Before use, PC was dissolved in IVM medium to prepare a stock solution, and the solution was stored in the dark at -20°C. After thawing, oocytes were used for viability and maturation calculations, MMP measurements, ROS detection, and early apoptosis analysis.

**Fig 1:**
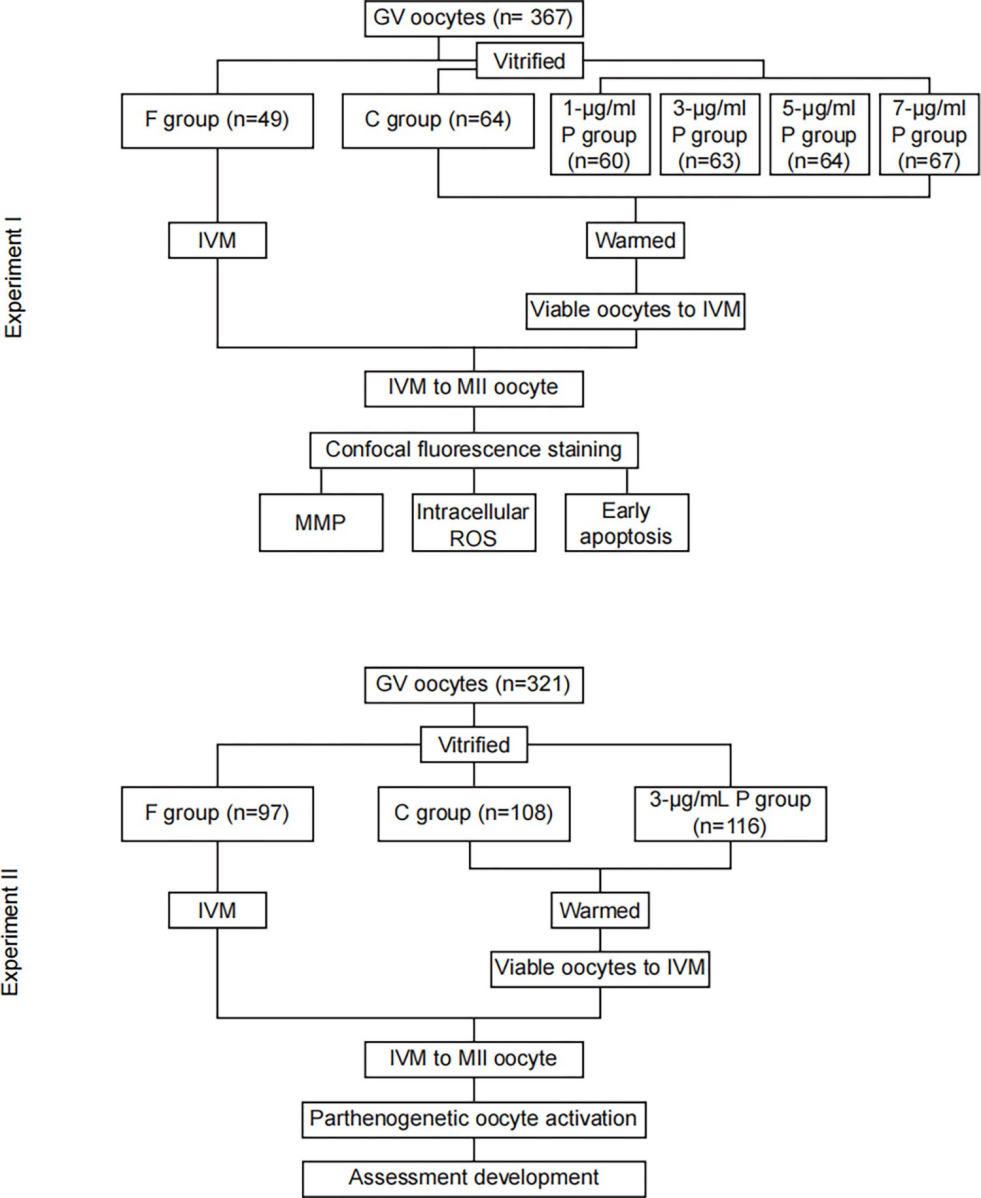
Flowchart of the experimental design.

Since 3 μg/mL PC was the optimal concentration for increasing the maturation rate after oocyte thawing, subsequent experiments were conducted with PC at this concentration by means of oocytes assigned to PC supplementation (P) group. In experiment II, 321 GV-stage oocytes were randomly assigned to the three groups to investigate the enhancing effect of PC on the cryopreservation of human oocytes. The developmental competence of vitrified-thawed oocytes from the C group, P group and F group was evaluated by using parthenogenetic activation and embryo *in vitro* culture. Fig 1 showed the experimental design scheme.

GV-stage oocytes were randomly divided into a fresh group without freezing treatment (F group), the vitrified control group without PC supplementation (C group), and the vitrified group with different concentrations of PC (P group). Following thawing, the recovered oocytes were subjected to IVM, MMP measurements, ROS evaluations, and early apoptosis assessments. A supplement of 3 μg/mL PC was employed as the P group for analysis of parthenogenetic and embryo developmental potential.

### Oocyte collection

The patients received standard ovarian stimulation utilizing either a long or short protocol. A gonadotropin-releasing hormone analog downregulates (Triptorelin Acetate, Ferring AG, Switzerland), the patients were stimulated with recombinant FSH (Gonal-F; Serono, Switzerland). When three or more follicles reached 18 mm in diameter, 6000~10000 IU human chorionic gonadotropin (hCG) (Lizhu, China) was administered. Ultrasound guided vaginal puncture was conducted for oocyte retrieval within 34–36 h after hCG injection. The cumulus-oocyte complexes (COCs) were isolated and cultured in fertilization medium at 37°C with 6% CO_2_ for 2~4 h. After denudation of COCs, the oocytes with a GV structure were used for subsequent experiments. Prior to cryopreservation, the oocytes were microscopically assessed for their structure to identify superior oocytes of suitable dimensions, intact zona pellucida, and intact membrane.

### Vitrification and warming of immature oocytes

Vitrification cooling and warming kits were used with the Cryotop^®^-open system (Kitazato, Japan). For cooling, oocytes were vitrified and warmed following a standard protocol: oocytes were gradually exposed to three ES: 1 minute in ES1, 2 minutes in ES2, and 6~10 min in ES3. Afterward, oocytes were transferred from ES3 to the VS for 30 s, loaded on the Cryotop devices and immersed in liquid nitrogen (LN2).

For warming, the Cryotop was removed from the LN2 within one second and fully immersed in a TS at 37°C by gentle agitation for 1 minute. The oocytes were then rinsed in DS for 3 minutes and transferred to WS1 and WS2 for 5 minutes. Next, the warmed oocytes were transferred to embryo culture medium with 20% (v/v) patient serum for 2 hours.

Survival of the oocyte was determined by the existence of a transparent, luminous, uniform cytoplasm along with an undamaged plasma membrane and zona pellucida. The ratio of surviving oocytes to frozen oocytes was used to calculate the survival rate.

### In vitro maturation

Commercial maturation medium (SAGE, USA) was used for oocyte IVM. Noncryopreserved oocytes or oocytes immediately after thawing were washed and cultured in 1 ml maturation medium supplemented with 75 mIU/ml FSH (Ferring, Germany), 75 mIU/ml LH (Ferring) and 20% (v/v) patient serum at 37°C in a humidified atmosphere comprising 6% CO_2_ and 5% O_2_ for 36 h. The maturity of the oocytes was assessed using an inverted microscope (IX-71, Olympus, Japan). Nuclear maturation of oocytes was defined as the presence of the first polar body (PB1). The remaining oocytes without PB1 were eliminated. The ratio of MII-stage oocytes to GV-stage oocytes subjected to maturation was used to calculate the maturation rate.

### Parthenogenetic oocyte activation and assessment of parthenogenetic embryonic development

Paffoni et al. outlined a chemical protocol for parthenogenetic activation. An evaluation was conducted to determine the developmental capacity of oocytes following the warming process [35]. MII-stage oocytes were exposed to G-IVF^™^ PLUS medium (Vitrolife, Sweden) containing 10 μmol/L ionomycin for 5 minutes in the dark. They were then washed twice and incubated for 3 h in G-1^™^ PLUS culture medium (Vitrolife) containing 2 mM 6-dimethylamino purine (6-DMAP) (Sigma Aldrich, USA). Subsequently, oocytes were washed three times in the same medium and cultured for 18 to 20 hours until activation was assessed. Oocytes that showed an elongated pronucleus and did not expel the second polar body were activated. The activation rate was defined as the ratio of activated oocytes to MII-stage oocytes subjected to activation.

After parthenogenetic activation, oocytes were washed twice and cultured in G-1^™^ PLUS medium for 3 days and G-2^™^ PLUS medium for another 2 days (114–116 hours after activation), followed by the assessment of blastocyst formation. According to the Istanbul Consensus [36], embryos are evaluated according to the developmental stage and morphological quality on the second day (42–44 hours after activation), the third day (66–68 hours after activation), the fourth day (92–94 hours after activation) and the fifth day (114–116 hours after activation). A Gardner and Schoolcraft classification system was used to classify blastocysts [37]. The ratio of cleavage to 2-cell parthenotes was used to calculate the developmental rate. The ratio of blastocyst to cleavage parthenotes was used as to calculate the blastocyst rate.

### ROS assay

To assess the level of ROS production, the observed MII oocytes were treated with 10 μM DCFH-DA (Beyotime Biotechnology Inc, China) and incubated at 37°C and 6% CO_2_ for 30 minutes in G-1^TM^ PLUS medium. After staining, the oocytes were transferred to cell imaging dishes. Fluorescence signals were measured using confocal microscopy (Nikon A1R, Japan). Photographs were analyzed using ImageJ (http://rsbweb.nih.gov/ij/) to measure the fluorescence intensity of the staining in each oocyte.

### Mitochondrial membrane potential (ΔΨm) detection

The measurement of MMP (ΔΨm) was conducted by utilizing the JC-10 mitochondrial inner membrane potential dye from Beyotime, following the instructions provided by the manufacturer. Oocytes were immersed in a working solution containing 10 μM JC-10 and incubated in the dark at a temperature of 37°C, with 6% CO_2_ and 5% O_2_ for a duration of 20 minutes. The evaluation of samples was conducted using confocal microscopy, following the aforementioned description. We calculated the ratio of red-to-green fluorescence intensity to determine MMPs in the oocytes.

### Annexin-V staining

The assessment of oocyte apoptosis was conducted by employing the Annexin-V-FITC Apoptosis Kit (Vazyme, China). Oocytes were treated with 195 μl of binding buffer, which included 5 μl of Annexin-V-FITC, and incubated in the dark for 30 minutes to be stained. Following three times washing, fluorescence signals were observed using confocal microscopy as previously explained. We detect the fluorescence intensity of each cytoplasmic membrane region and calculate the average fluorescence intensity of each oocyte. Oocytes above this intensity are considered to have undergone early apoptosis, while oocytes below this intensity are considered to have not undergone apoptosis.

### Statistical analysis

The data are presented as the mean ± S.D. Statistical analysis was based on the data from at least three biologically independent replicates. Staining was repeated at least three times, and representative micrographs from similar results are shown. Statistical comparisons were made using an analysis of variance (ANOVA), and differences between each group were assessed using Newman‒Keuls multiple comparisons post hoc tests. For the analysis of rates of maturation, activation, development, and blastocyst formation, chi-squared tests in Microsoft Excel software (Microsoft Corporation, USA) were used. The level of statistical significance was set at *P*<0.05.

## Results

### PC facilitates the survival and maturation of vitrified GV-stage oocytes

We first evaluated the effect of PC on cryopreserved oocyte survival and maturation. Table 1 illustrates that the survival rates of the C group and the P group did not differ significantly (*P*>0.05). However, when evaluating the percentage of maturation among the oocytes that survived, it was observed that the rate of maturation in the C group was significantly less than that of the F group. Significantly, supplementation with 3 μg/mL PC increased the maturation rate, which was much higher than that of the C group (*P*<0.05) and comparable to that of the F group, indicating that this is a suitable concentration and can facilitate the maturation of vitrified human GV-stage oocytes.

**Table 1:** Comparisons of the survival and maturation rates among the groups.

		Vitrification group				
	F group (n = 49)	C group (n = 64)	P group (n = 254)			
	N/A	N/A	**1 μg/mL (n = 60)**	**3 μg/mL** **(n = 63)**	**5 μg/mL (n = 64)**	**7 μg/mL (n = 67)**
**No. of surviving oocytes/total oocytes (%)**	N/A	59/64 (92.2±5.1%)^a^	55/60 (91.7±3.5%)^a^	58/63 (92.1±2.5%)^a^	58/64 (90.6±4.9%)^a^	59/67 (88.1±7.1%)^a^
**No. of matured oocytes/surviving oocytes (%)**	43/49 (87.8±1.1%)^a^	41/59 (69.5±0.9%)^b^	39/55 (70.9±4.2%)^b^	50/58 (86.2±7.6%)^a^	40/58 (68.9±7.9%)^b^	41/59 (69.5±6.4%)^b^

Within the same line, percentages without the same superscript indicate statistically significant differences (*P*<0.05).

### Morphology of vitrified-warmed GV-stage oocytes treated with different concentrations of PC

**Fig 2:**
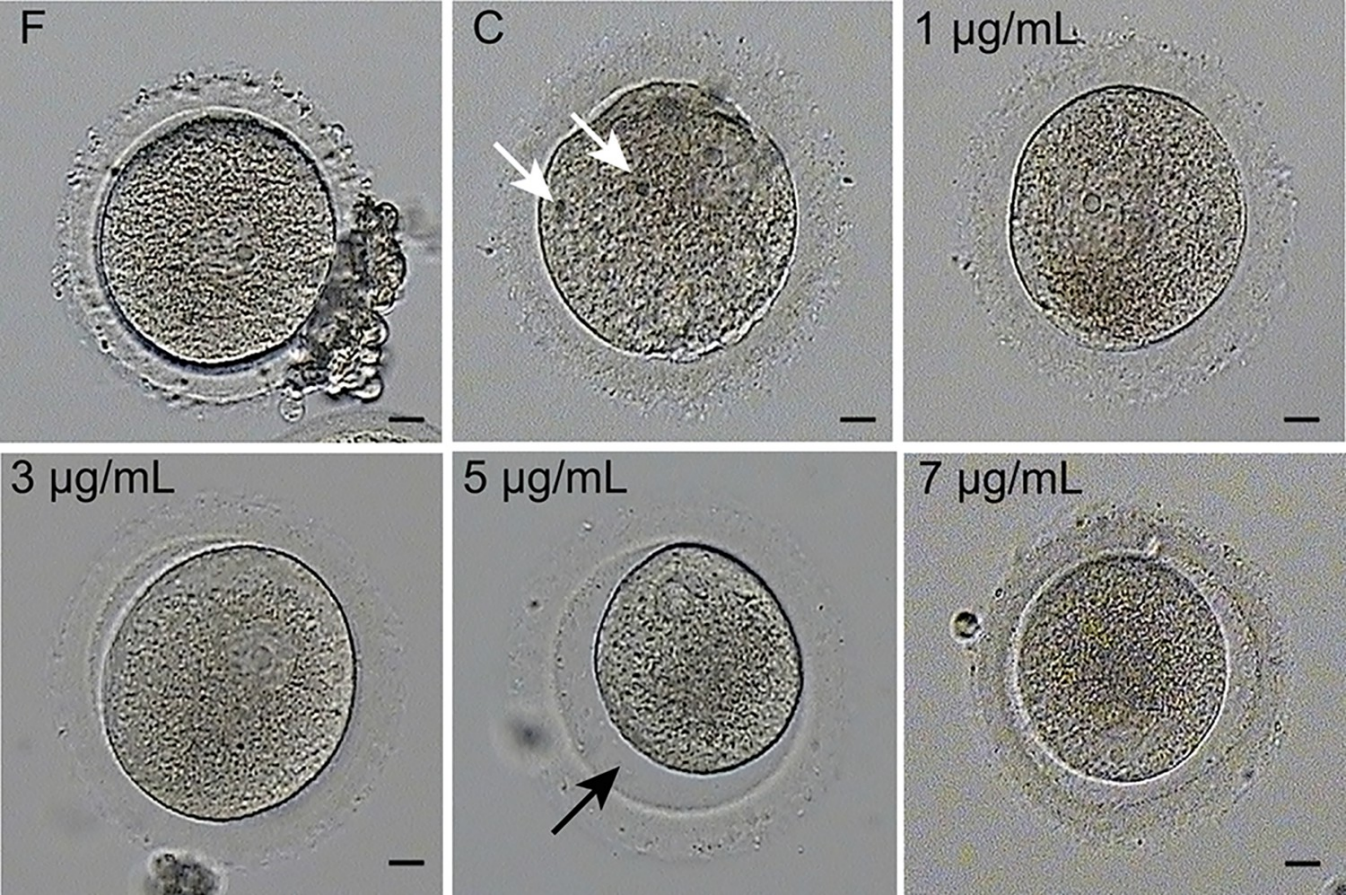
Morphological analysis of GV-stage oocytes after vitrification and warming. F: Fresh oocyte without freezing; C: Oocytes recovered from freezing without PC supplementation; P: Oocytes retrieved from freezing with different concentrations (1 μg/mL, 3 μg/mL, 5 μg/mL, and 7 μg/mL) of PC. Morphological observations were conducted by optical microscopy. The white arrow indicates granulation clusters, and the black arrow indicates a large perivitelline space in the oocyte, Bar = 10 μm.

Oocyte morphology was observed before or after cryopreservation with different concentrations of PC. As shown in Fig 2, fresh oocytes without cryopreservation treatment in the F group maintained regular morphology with a uniform cytoplasm and regular perivitelline space. In contrast, oocytes subjected to vitrification in the C group exhibited irregular shapes characterized by clusters of granulation within the cytoplasm or a significant perivitelline space. Interestingly, supplementation with 3 μg/mL PC partially reversed the abnormal morphology of the cryopreserved oocytes, similar to that of the fresh oocytes. Thus, PC at a concentration of 3 μg/mL was used to evaluate the oocytes in the P group.

### PC protects mitochondrial function and attenuates oxidative stress and early apoptosis in vitrified GV-stage oocytes after IVM

**Fig 3:**
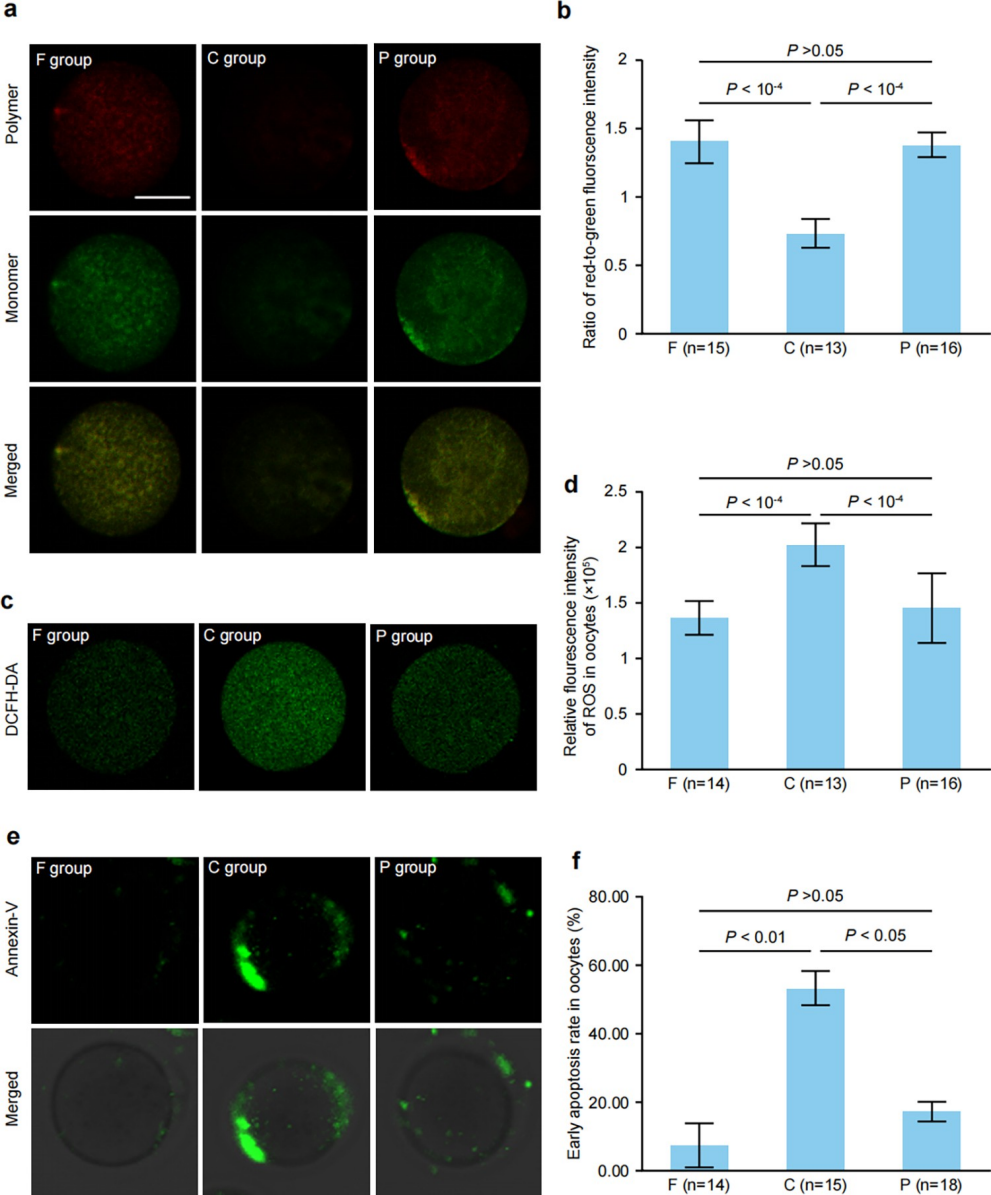
Effect of PC treatment on mitochondrial membrane potential (MMP), intracellular ROS and early apoptosis in vitrified-warmed GV-stage oocytes after IVM. F: Fresh oocyte without freezing; C: Oocytes recovered from freezing without PC supplementation; P: Oocytes retrieved from freezing with supplementation of 3 μg/mL PC. The number of detected oocytes for each group is noted in brackets. (a) Representative images of mitochondrial membrane potential after fluorescence staining with JC-10. Red, highΔΨm; Green, lowΔΨm. Bar = 50 μm. (b) JC-10 fluorescence intensity was quantified. Data are expressed as the mean ± S.D. *P* values are labeled for each comparison. (c) Representative images of intracellular ROS levels after fluorescence staining with DCHF-DA. Green, FITC, Bar = 50 μm. (d) DCHF-DA fluorescence intensity was quantified. Data are expressed as the mean ± S.D. *P* values are indicated for each comparison. (e) Representative images of early apoptosis in oocytes after fluorescence staining with Annexin-V. An unambiguous green membrane signal characterizes oocytes undergoing early apoptosis; Bar = 50 μm. (f) Data are expressed as the mean ± S.D. *P* values are indicated for each comparison.

As mitochondrial function is closely associated with oocyte quality, MMP was used an indicator of mitochondrial function and examined by measuring the relative levels of red-to-green fluorescence emission by means of the JC-10 fluorescent dye. As shown in Fig 3A and 3B, compared with the C groups, PC treatment significantly increased MMP levels (*P*<10^−4^). Conversely, vitrification minimized the function of mitochondria, as indicated by the significant decrease in MMP (*P*<10^−4^).

ROS levels can indicate oocyte oxidative stress. To assess the impact of vitrification and PC on oxidative stress within oocytes, we measured DCFH-DA fluorescence intensity as an indicator of intracellular ROS levels. As shown in Fig 3C, MII oocytes in the F group had much lower fluorescence intensity than those in the C group (*P*<10^−4^). Conversely, PC supplementation minimized the production of ROS to a level similar to that in the F group (*P*>0.05) (Fig 3C and 3D). These results indicate that PC administration can partially inhibit the production of ROS in oocytes during vitrification.

Higher levels of intracellular ROS are associated with significant early apoptosis in cells. To ascertain the fraction of oocytes subject to early apoptotic events, we conducted Annexin-V staining. In the F group, only the oocyte zona pellucida displayed a faint green fluorescence signal from Annexin-V. However, in the C group, significantly higher positive signal intensities were detected in the oocyte membrane and zona pellucida. PC treatment significantly inhibited early oocyte apoptosis (Fig 3E). We calculated the percentage of oocytes with early apoptosis. The data showed that only 7.1% of the oocytes in the F group had a positive signal, and this was comparable to the percentage in the P group (16.7%) and much lower than that in the C group (53.3%) (Fig 3F). The results suggest that freezing oocytes leads to a higher level of early apoptosis, while PC treatment successfully prevents this worsening.

### PC protects the developmental competence of GV-stage oocytes after cryopreservation and IVM

**Fig 4:**
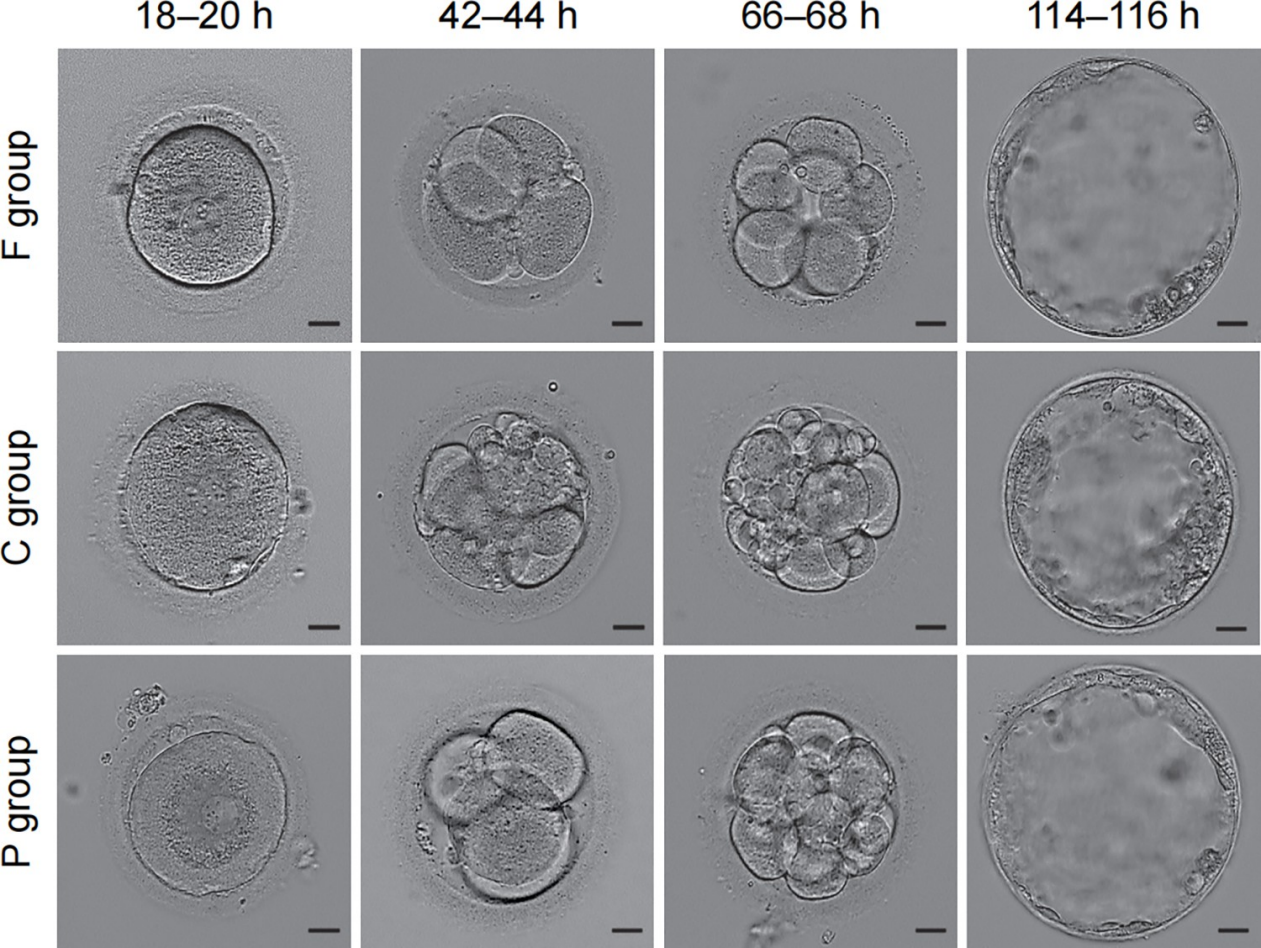
Embryo morphology development from oocytes after different treatments. Representative images consisting of pronuclear oocytes (first row), 4-cell embryos (second row), 8-cell embryos (third row), and blastocysts (fourth row). Bar = 10 μm.

Parthenogenetic activation was performed with the MII oocyte after the IVM procedure. Activated oocytes that developed to the pronuclear stages at 18–20 hours, cleaved at 42–44 and 66–68 hours, and formed blastocysts at 114–116 hours were recorded to evaluate the embryo developmental potential (Table 2). The morphology of embryos developed to the referred stages for each group is shown in Fig 4.

**Table 2:** Comparison of the developmental rates of the early parthenotes among the groups.

	F group (n = 86)	Vitrification group	
		C (n = 69)	P (n = 90)
**No. of activated oocytes (%)** **18–20 h after activation**	74 (86.0±5.1%)^a^	51 (73.9±9.0%)^a^	68 (75.6±9.1%)^a^
**No. of cleaved oocytes (%)** **42–44 h after activation**	60 (81.1±4.5%)^a^	40 (78.4±5.5%)^a^	52 (76.5±5.5%)^a^
**No. of cleaved oocytes (%)** **66–68 h after activation**	43 (58.1±5.7%)^a^	19 (37.3±3.9%)^b^	39 (57.4±7.0%)^a^
**No. of blastocysts (%)** **114–116 h after activation**	12 (27.9±6.0%)^a^	1 (5.3±6.4%)^b^	9 (23.1±10.7%)^ab^

Within the same line, percentages without the same superscript indicate statistically significant differences (*P*<0.05).

There were no significant differences in the percentage of oocyte activation among the three groups (P>0.05). Additionally, there were no significant differences among the three groups when the percent of cleaved embryos at 42–44 hours was compared (*P*>0.05). However, when comparing the percent of cleaved embryos at 66–68 hours, oocytes in the F and P groups had much higher potential than those in the C group (*P*<0.05). Regarding blastocyst formation at 114–116 hours, oocytes in the F group had the highest percentage compared to those in the C group (*P*<0.05). PC supplementation increased the developmental potential of oocytes to the blastocyst stage. Nevertheless, no statistically significant difference was found between the P and C groups (*P*>0.05) (Table 2). These results indicate that PC could improve the developmental potential of vitrified oocytes after parthenogenetic activation.

Furthermore, group comparisons of the numbers of blastomeres at 42–44 hours and 66–68 hours after activation were performed. As shown in Table 3, the number of blastomeres was comparable for each group at 42–44 hours and 66–68 hours after activation (*P*>0.05), indicating that vitrification itself or PC supplementation does not affect the cell division rate.

**Table 3:** Comparison of the number of blastomeres from parthenotes among the groups.

Time after activation	F group	Vitrification group	
		C	P
**42–44 h**	3.7±0.7^a^	3.6±0.7^a^	3.5±0.7^a^
**66–68 h**	6.8±1.1^a^	6.2±1.0^a^	6.4±1.0^a^

Within the same line, percentages without the same superscript indicate statistically significant differences (*P*<0.05).

We next checked whether vitrification and PC affected embryo quality. As shown in Table 4, 71.7% of grade I-II parthenotes were at 42–44 hours after activation in the F group, which is much higher than the percentage in the C group (47.5%, *P*<0.05). Notably, when PC was used during vitrification and subsequent oocyte activation and early embryo development, a higher percentage of grade I-II parthenotes was obtained, comparable to that in the F group (*P*>0.05). Similar results were obtained when parthenotes developed 66–68 hours after activation. In the P group, 61.5% of parthenotes were classified into grades I-II, which is much higher than that in the C group (31.6%, *P*<0.05) and comparable to that in the F group (60.5%, *P*>0.05) (Table 4).

**Table 4:** Comparison of parthenote quality among the groups.

	F group	Vitrification group	
		C	P
**Grade I-II parthenotes (%)** **42–44 h**	43 (71.7±3.2%)^a^	19 (47.5±8.5%)^b^	36 (69.2±9.5%)^a^
**Grade I-II parthenotes (%)** **66–68 h**	26 (60.5±6.4%)^a^	6 (31.6±10.1%)^b^	24 (61.5±9.8%)^a^

Within the same line, percentages without the same superscript indicate statistically significant differences (*P*<0.05).

## Discussion

The cryopreservation of human immature oocytes presents distinct advantages over their mature counterparts. Since the chromosomes remain within the nucleus, immature oocytes may circumvent the problem of spindle damage that frequently occurs in MII oocytes throughout the cryopreservation process [38,39]. Moreover, the cryopreservation of immature oocytes can help safeguard fertility in tumor patients prior to undergoing chemotherapy or radiation therapy, without postponing their treatment or facing risks associated with inducing oocyte maturation[39]. Nonetheless, there are also drawbacks to the cryopreservation of human immature oocytes. Immature oocytes that are frozen, thawed, and then subjected to IVM before insemination demonstrate reduced rates of fertilization and cell division compared to mature oocytes. Therefore, the cryopreservation of mature oocytes is still more efficient than that of immature oocytes [40].

In this study, we tested the effects of PC on the vitrification of human GV-stage oocytes by evaluating survival, maturation, and parthenote development. The protective impact of PC in the vitrification led to an enhancement in the potential for maturation, as well as betterment in embryo cleavage and growth. Clearly, this provides an effective approach for certain cancer patients to augment the pool of fully developed ova in those who have an increased likelihood of suffering from premature ovarian failure or in cases where controlled ovarian stimulation is inadvisable.

Recent evidence indicates that PC functions as an antioxidant, enhancing the quality of porcine oocytes and their subsequent developmental competence in embryos [34]. Hence, the primary objective of our current research was to identify the optimal concentration of PC that would enhance the maturation rate of human oocytes at the GV-stage after cryopreservation. It was observed that the survival rate of GV-stage oocytes in the vitrification group, which were treated with different levels of PC, markedly improved, likely as a result of the unique cellular architecture present at the GV stage. The next question is whether maturation potential can be rescued due to the presence of PC during the cryopreservation process. Our study demonstrates that 3 μg/mL PC can significantly improve the maturation rate of vitrified oocytes after the IVM procedure. This implies that PC supports the metabolic environment necessary for immature oocytes to reach maturation during IVM culture. These results also indicate that an effective PC IVM program has the benefit of enabling the reuse of GV-stage oocytes from the COH cycle.

Studies have shown that heightened oxidative stress could potentially be the cause of diminished quality in vitrified-warmed oocytes [41]. The accumulation of ROS [42] is a major cause of oxidative stress, potentially playing a critical role in damaging proteins, DNA, lipids, and the cell membrane [43], thereby disrupting the structure and function of oocytes. Nevertheless, optimizing culture conditions is a multifaceted endeavor that hinges on carefully choosing an appropriate antioxidant and precisely calibrating its concentration to support oocyte in resisting damage caused by vitrified-warmed. The ultimate outcome of incorporating antioxidants should be to ensure that the levels of ROS remain within the normal range and to preserve the equilibrium of redox reactions. In our study, the effect on embryo morphology improvement was not achieved in the 1 μg/mL, 5 μg/mL, and 7 μg/mL PC subgroups. Notably, the maturation rate in the 3 μg/mL PC subgroup increased to a level close to that of the F group. According to previous research, it has been shown that meiotic resumption requires the presence of physiological levels of ROS [28,44]. In the present study, we propose that the addition of 3 μg/mL PC can counteract the additional ROS induced by *in vitro* manipulation. In contrast, supplementing other PC concentrations may disrupt the balance, resulting in a lower maturation rate. The cultivation of oocytes outside a living organism does not provide a natural antioxidant system as found *in vivo*, potentially leading to an overproduction of ROS and subsequent oxidative stress [45]. Antioxidants in the culture medium may serve as scavengers of ROS.

Under physiological conditions, ROS play a crucial role in the nuclear maturation of oocytes. However, when excessive production of ROS occurs, it induces oxidative stress, compromises mitochondrial function, and obstructs the progression of oocyte development. Maintaining mitochondrial function requires a delicate balance between the generation of ROS and the capacity of antioxidants [46]. In order to investigate how PC enhances the quality of vitrified-warmed oocytes, we assessed the levels of ROS using the intensity of DCFH-DA fluorescence. The results showed that the vitrified-warmed oocytes in the PC group with a concentration of 3 μg/mL exhibited reduced levels of intracellular ROS compared to the oocytes in the C group. This confirmed that PC could scavenge the excess ROS to a certain degree.

Considering that elevated ROS levels trigger premature oocyte apoptosis [47], we utilized Annexin-V staining to assess the percentage of oocytes undergoing early apoptotic processes. In comparison to the oocytes of the C group, a notable reduction in early apoptosis was observed in the oocytes of the PC group with a concentration of 3 μg/mL. This finding was consistent with the hypothesis that PC minimizing fragmentation and degeneration is likely attributable to its ability to suppress the production of ROS.

Other research suggests that oocyte apoptosis is connected to an overabundance of ROS and heightened dysfunction within the cell and mitochondria [28,48,49], additionally, the decline in oocyte developmental competence caused by stress is linked to changes in mitochondrial function [50,51]. Hence, we measured the MMP. The results indicated that the vitrified-warmed oocytes obtained from the P group exhibited a higher MMP compared to those from the C group. The findings suggest that PC has the ability to successfully safeguard the mitochondrial function of cryopreserved oocytes against oxidative stress by diminishing levels of ROS.

Reportedly, PC has the potential to enhance oocyte IVM and facilitate the early growth of embryos in pigs [52]. However, additional data is required to determine if PC enhances the developmental capacity of GV-stage human oocytes obtained from the COH cycle. Since safety concerns prohibit the use of PC in fertilizing human oocytes for research, parthenogenetic activation is utilized to evaluate the impact of PC on the embryo development. Our findings demonstrate that including PC in the cryoprotectants and culture medium results in a higher potential and improved embryo cleavage and development. The P group demonstrated a significantly higher rate of forming high-quality blastocysts in comparison to the group referred to as C. Hence, the results indicate that applying PC treatment may efficiently maintain the mitochondrial oxidative phosphorylation activity within oocytes by preventing stress during the vitrification-warming process and IVM culture, thereby aiding in the advancement of subsequent embryonic development.

The low rate of blastocyst formation observed in this study could likely be due to the embryos being created through IVM and parthenogenesis at GV-stage, a process which excluded the participation of sperm for fertilization.

The reduced occurrence of blastocyst growth observed in this study can likely be ascribed to embryos originating from GV-stage oocytes that have undergone IVM and parthenogenetic activation, which did not involve the use of sperm for fertilization. In comparison, when fully matured MII vitrified oocytes are used, there is a high production of quality blastocysts on the fifth day following ICSI, which stands in stark contrast (S1 Table).

## Conclusions

**Fig 5:**
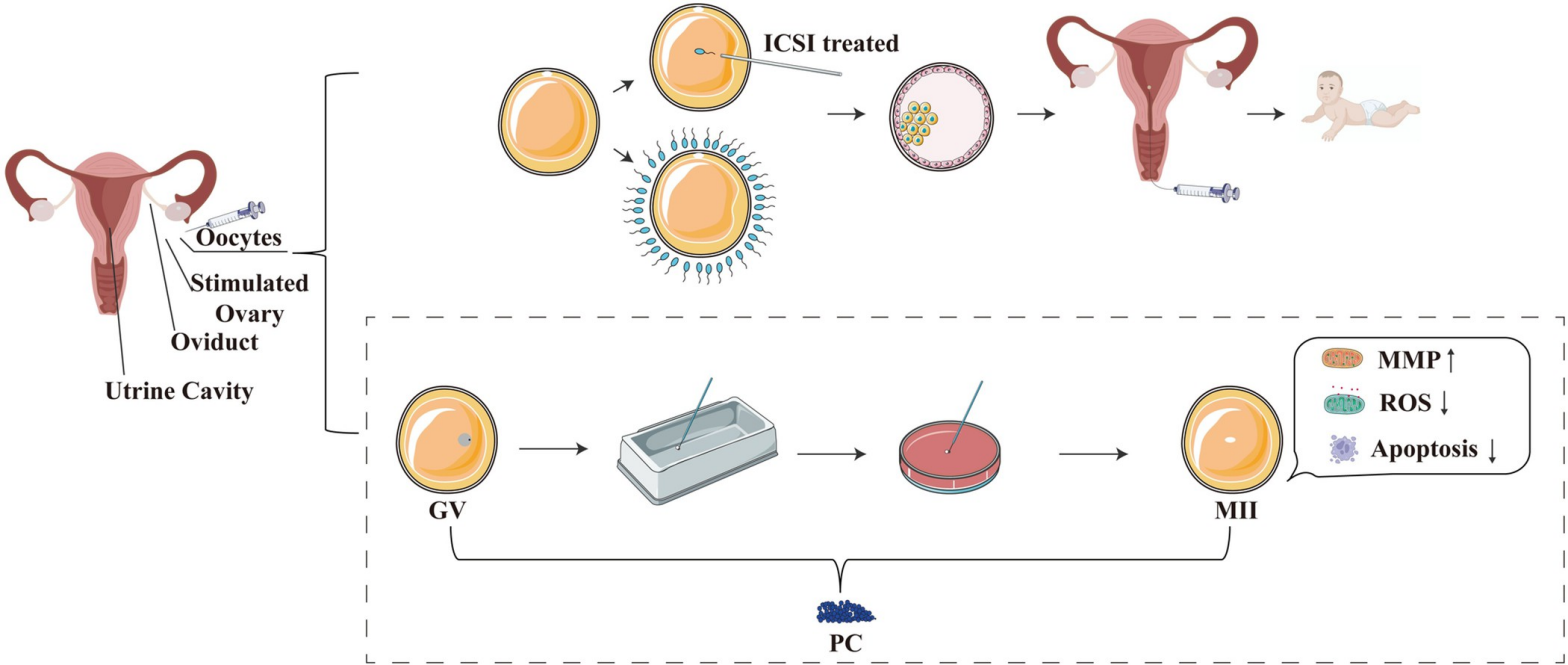
Reuse of GV oocytes retrieved from the COH cycle through PC-treatment. IVM culture of GV stage oocytes recovered from the COH cycle was supplemented with PC, followed by parthenogenetic activation and embryo culture to form blastocysts. PC protected oxidative phosphorylation pathways, mitochondrial function, increased MMP, and decreased intracellular ROS and apoptosis rates (as shown in the dashed box).

In conclusion, we have shown that the presence of PCs in both the VS and culture medium can significantly enhance the efficacy of cryopreservation in human GV-stage oocytes. Suppressing oxidative stress is likely to enhance the developmental competence of cryopreserved oocytes. The PC directly decreases levels of ROS in oocytes in order to safeguard mitochondrial function, thereby preventing the worsening of early apoptosis (Fig 5).

## Supporting information

S1 Table The embryo development rate after ICSI fertilization of freezing-warmed MII oocytes. (DOCX)

S1 Data Raw data for Tables 1–4, S1 Table and Fig 3B, 3D and 3F. (XLSX)
