# Supplementary material for: C-phycocyanin improves the developmental potential of cryopreserved human oocytes by minimizing ROS production and cell apoptosis
Source: PLoS One. 2024 Apr 1;19(4):e0300538. doi: 10.1371/journal.pone.0300538 (PMC10984518; doi:10.1371/journal.pone.0300538)
Supplement: S1 Table — (DOCX) [file pone.0300538.s001.docx]

**Supporting information**

**S1 Table. The embryo development rate after ICSI fertilization of freezed-warmed MII oocytes .**

|  | **MII oocytes (n=85)** |
| --- | --- |
|  |  |
| No. of activated oocytes (%) after ICSI | 70 (82.3±3.4%) |
| No. of cleaved oocytes (%) after ICSI | 68 (97.1.0±2.2%) |
| No. of blastocysts (%) after ICSI | 32 (47.1±2.3%) |
| No. of High-quality blastocysts (%) | 20 (29.4±3.3%) |
